# P-240. HIV Provider Perspectives on Anal Cancer Screening: a National Survey

**DOI:** 10.1093/ofid/ofaf695.462

**Published:** 2026-01-11

**Authors:** Maria C Geba, Hannah Pambianchi, Divya Kalluri, Tania Thomas

**Affiliations:** University of Virginia, Richmond, VA; University of Virginia, Richmond, VA; Donald and Barbara Zucker School of Medicine at Hofstra/Northwell, Uniondale, New York; University of Virginia, Richmond, VA

## Abstract

**Background:**

There is growing evidence that ablation of high-grade anal dysplasia prevents the development of anal cancer. Despite this, anal cancer screening uptake is lagging in many clinical settings. We conducted a national survey study for HIV providers to assess beliefs about anal cancer screening, barriers to screening and needs for implementing a screening program in their clinic.

**Methods:**

An 18-question survey was built on REDCap and distributed among medical providers that care for adults with HIV in the United States. The online survey was distributed through an HIV society listserv, social media platforms and direct messaging of academic infectious disease program directors.

**Results:**

Two hundred and sixty-eight participants completed a survey, which resulted in a 2.8% response rate. Of those, 227 participants were included in further analysis. Sixty two percent (n=141) were women, 63% were attendings (n=144) and 60% were infectious disease specialists (n=136). Forty participants did not offer anal cancer screening (18%). Of the 187 participants that offered screening, 81% (n=152) performed their own anal pap smears and 72% (n=120) referred out for anal biopsies. Of those that offered anal cancer screening, the most common challenge was lack of interest from patients (30%, n=57). Among those that did not offer anal cancer screening, 98% (n=39) stated they were motivated or fairly motivated to offer screening if they had an established pathway. And 75% (n=30) wanted to receive training for anal Pap smears. The most common barrier to screening in this group was lack of referral options (53%, n=21) and the most pressing need in order to start a screening program was establishing referral pathways (53%, n=21).

**Conclusion:**

There are several barriers that need to be addressed in order to empower HIV providers to screen for anal cancer. For those without an established anal cancer screening program, the vast majority would be motivated to screen their patients if they have a screening program in place. The most pressing needs for this group are establishing referral pathways to specialists and increasing training opportunities for themselves or staff.

**Disclosures:**

All Authors: No reported disclosures

